# ^18^F-FAPI-74 PET/CT findings of an inflammatory branchial cleft cyst mimicking cervical metastasis from carcinoma of unknown primary: A case report

**DOI:** 10.1016/j.radcr.2026.01.064

**Published:** 2026-02-13

**Authors:** Seiji Oyagi, Tomohiko Yamane, Takuya Yamamoto, Norio Yamamoto, Hara Shigeo, Michio Senda, Masahiro Kikuchi

**Affiliations:** a Department of Otolaryngology, Head and neck surgery, Kobe City Medical Center General Hospital, 2-1-1 Minatojima Minamimachi, Chuo-ku, Kobe City, Hyogo 650-0047, Japan; b Department of Molecular Imaging Research, Kobe City Medical Center General Hospital, 2-1-1 Minatojima-minamimachi, Chuo-ku, Kobe City, Hyogo 650-0047, Japan; c Department of Diagnostic Pathology, Kobe City Medical Center General Hospital, 2-1-1 Minatojima-minamimachi, Chuo-ku, Kobe City, Hyogo 650-0047, Japan

**Keywords:** Branchial cleft cyst, Head and neck neoplasms, Diagnostic imaging, Positoron-emission tomography

## Abstract

Fibroblast-activation protein inhibitor (FAPI) positron-emission tomography (PET) is emerging as a promising alternative to ^18^F-fluorodeoxyglucose (FDG) PET for head-and-neck cancer. Herein, we describe the case of a 51-year-old man with a cystic level-II neck mass that was intensely FDG-avid and even more avid on ^18^F-FAPI-74 PET. FDG-PET was performed as standard imaging modality to search for the primary tumor, while ^18^F-FAPI-74 PET was conducted as part of a clinical trial to detect potential occult lesions missed by FDG. While cytology suggested metastatic squamous-cell carcinoma and FDG PET revealed asymmetric tonsillar uptake, raising suspicion of an occult oropharyngeal primary tumor, ^18^F-FAPI-74 PET demonstrated an absence of tracer uptake in Waldeyer’s ring. The patient subsequently underwent ipsilateral palatine-tonsillectomy, lingual-tonsil biopsy, and radical neck dissection. Histopathological examination definitively excluded malignancy, identifying the lesion as an inflamed branchial cleft cyst with marked fibroblast proliferation, and confirmed that both tonsils were benign. Consequently, the preoperative cytology suggesting metastatic squamous cell carcinoma was determined to be a false-positive finding. This case underscores the potential of ^18^F-FAPI-74 PET to exclude tonsillar primaries in carcinomas of unknown primary origin, while highlighting that fibrotic inflammation can cause false-positive uptake.

## Introduction

Cervical lymph-node metastasis from carcinoma of unknown primary (CUP) remains a clinical challenge, for which accurate imaging is crucial in locating hidden primary tumors prior to treatment. ^18^F-fluorodeoxyglucose (FDG) positron-emission tomography (PET)/Computed tomography (CT) improves the detection rate of occult mucosal primaries, and is therefore recommended in international guidelines [1,2]. However, physiologic or inflammatory FDG uptake in Waldeyer’s ring often masks small oropharyngeal tumors, limiting diagnostic confidence.

Fibroblast-activation protein inhibitors (FAPI) are small- molecular tracers that bind with high affinity to fibroblast-activation proteins on cancer-associated fibroblasts, while showing minimal uptake in most normal tissues. This ultra-low background results in markedly higher tumor-to-background ratios (TBR) than FDG tracing across a range of malignancies. FAPI tracers can be radiolabeled with either ^68^Ga or ^18^F, enabling both generator- and cyclotron-based production platforms, while preserving high target affinity [3,4].

Because FAP is also upregulated in fibrotic or chronically inflamed tissues, tracer uptake can occur in benign lesions rich in activated fibroblasts, whereas nonfibrotic lymphoid inflammation typically shows minimal FAPI activity [[5], [6], [7]]. Herein, we report a case in which an inflamed branchial cleft cyst exhibited intense uptake on both FDG and ^18^F-FAPI-74 PET/CT, closely mimicking nodal metastasis and prompting extensive surgery. However, on retrospection, the absence of FAPI uptake in the Waldeyer’s ring indicated that tonsillar resection may have been avoidable, highlighting both the diagnostic advantage and potential pitfall of FAPI imaging when CUP is suspected.

## Case report

The patient was a 51-year-old man who noticed a painful swelling below the left ear which showed gradual enlargement over 14 months. His medical history included atopic dermatitis. He was a social drinker, but had stopped smoking 20 years prior. Flexible nasopharyngolaryngoscopy revealed no mucosal abnormalities.

**Fig. 1:**
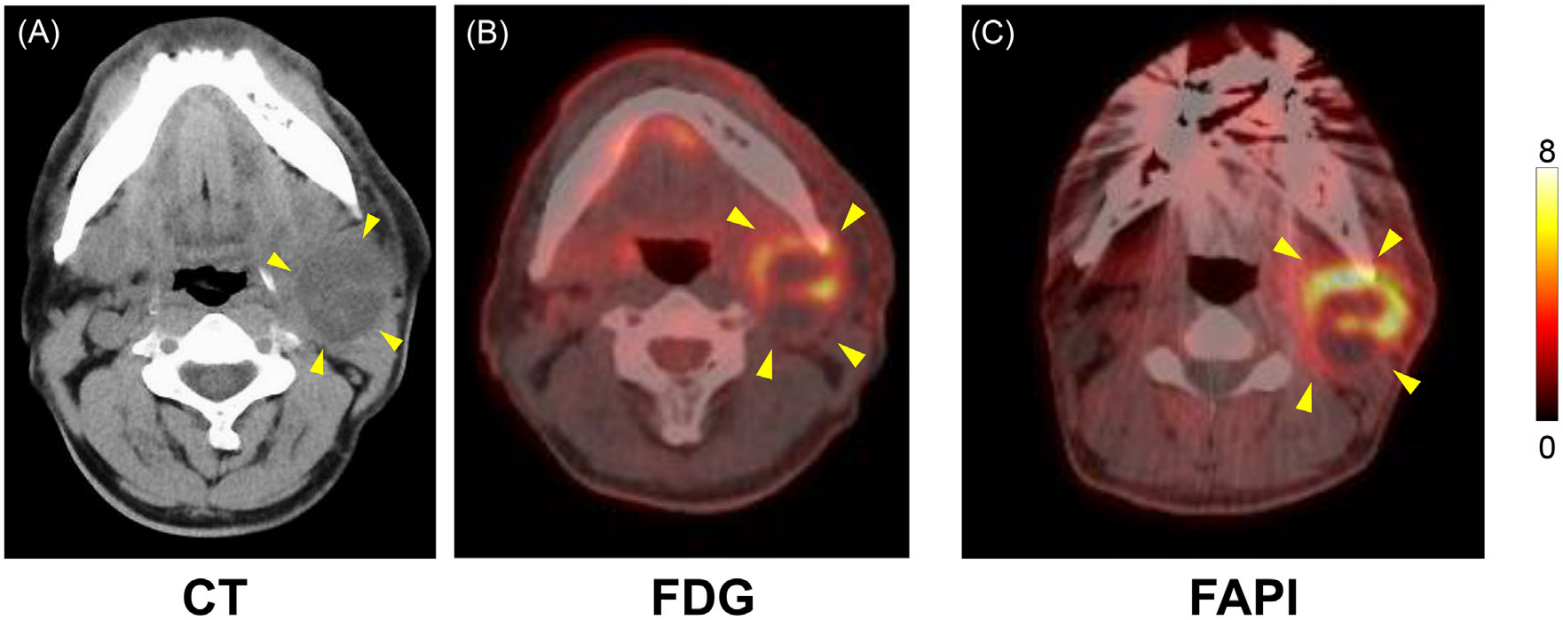
Multimodality imaging of the left level-II mass. (A) Axial noncontrast CT showing a 40 mm cystic–solid lesion with ill-defined margins (arrowheads). (B) Axial FDG PET/CT demonstrating intense tracer uptake (SUVmax 10.2) and (C) Axial ^18^F-FAPI-74 PET/CT at the identical slice level showing a similarly intense uptake (SUVmax 15.4). Color scale range 0-8, adjusted for visual comparison (actual SUVmax 15.4).

**Fig. 2:**
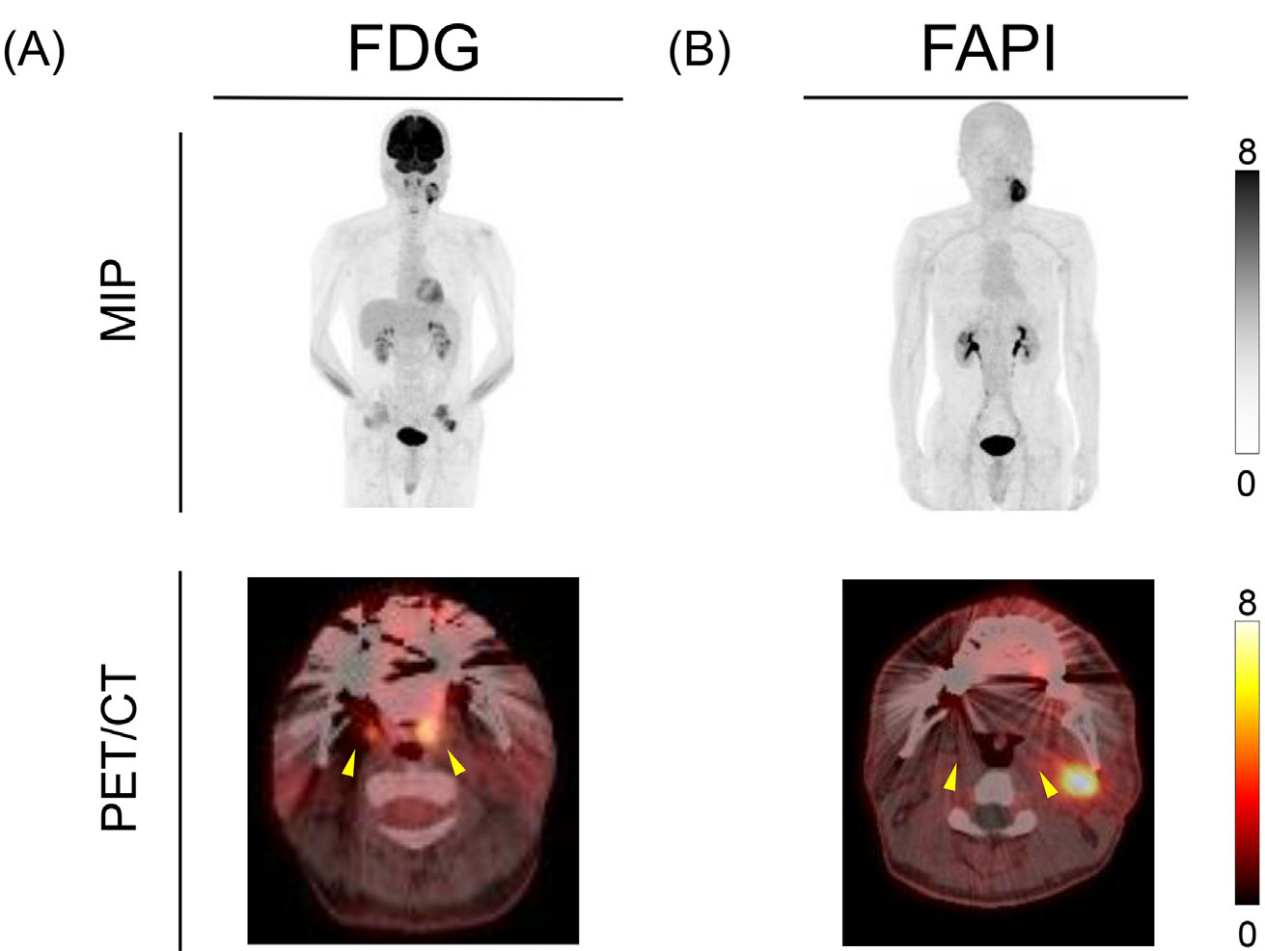
Evaluation of Waldeyer’s ring. (A) FDG PET/CT demonstrating asymmetric uptake with stronger accumulation in the left palatine tonsil (left > right), raising suspicion of an occult primary tumor and (B) ^18^F-FAPI-74 PET/CT showing no tracer accumulation in either tonsil, thus supporting the absence of an oropharyngeal primary. Arrowheads indicate the palatine tonsils. Color scale range 0-8, adjusted for visual comparison (actual SUVmax 15.4).

Noncontrast CT revealed a 40 × 40 mm cystic–solid mass with ill-defined margins in the left level-II region (Fig. 1A). FDG PET/CT revealed intense uptake within the lesion (SUVmax, 10.2) (Fig. 1B). The SUVmax of the palatine tonsils revealed no substantial laterality (left 6.3 vs right 6.0); however, visually asymmetric FDG uptake suggested the possibility of an occult primary in the left palatine tonsillar region (Fig. 2A). ^18^F-FAPI-74 PET/CT demonstrated a tracer-uptake pattern that closely paralleled the FDG findings; intense accumulation was again limited to the level-II mass (SUVmax 15.4), confirming the malignant impression, whereas no physiological activity was present in the Waldeyer’s ring, thereby highlighting a key discordance in the tonsillar region (Fig. 1C and 2B). In ultrasound-guided fine needle aspiration cytology (FNAC), keratinous material within the cyst was interpreted as Orange G-positive atypical cells, resulting in a diagnosis of suspected metastatic squamous cell carcinoma.

Imaging and cytology strongly indicated cervical lymph-node metastasis of unknown primary origin; therefore, the patient underwent left radical neck dissection combined with ipsilateral palatine-tonsillectomy and lingual-tonsil biopsy. Intraoperatively, neither tonsil displayed any gross tumors. The cervical mass was densely adherent to the platysma, posterior belly of the digastric muscle, sternocleidomastoid muscle, and internal and external jugular veins. All of the involved tissues were resected *en bloc*. The lesion extended into the parapharyngeal space, necessitating additional resection of the styloglossus and stylopharyngeal muscles. This resection resulted in a pharyngocervical fistula that was closed in a staged manner using a pectoralis major myocutaneous flap.

**Fig. 3:**
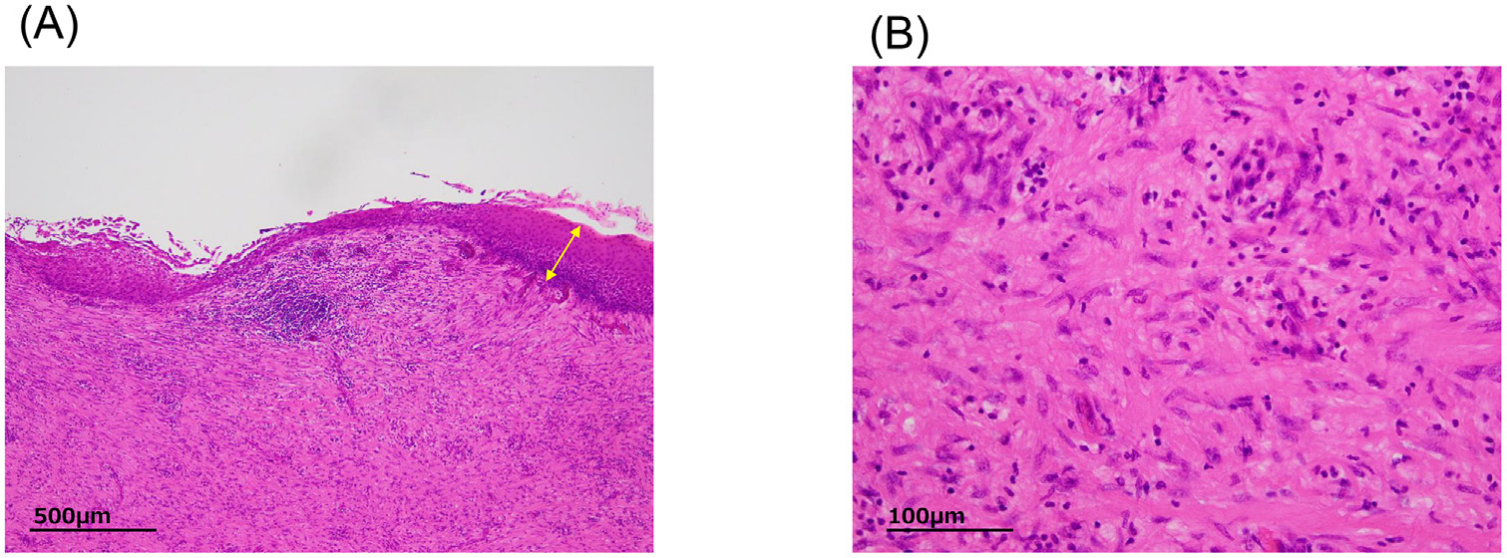
Histopathology of the neck mass. (A) Hematoxylin and eosin-stained section showing a cyst wall lined with keratinizing squamous epithelium without atypia (double-headed arrow) and (B) Higher-magnification view showing dense lymphocytic infiltration and activated fibroblasts in the pericystic stroma.

Macroscopic examination of the neck specimen revealed a unilocular cyst. Microscopically, the cyst wall was lined with keratinized squamous epithelium without atypia (Fig. 3A). Dense lymphocytic infiltration and reactive fibroblast proliferation were observed in the pericystic stroma (Fig. 3B). Immunohistochemical staining for p16 yielded negative results. Both the palatine and lingual tonsils were free of malignancy, confirming the presence of an inflamed branchial cleft cyst.

## Discussion

Physiological FDG uptake in the Waldeyer’s ring frequently masks oropharyngeal primaries in patients with CUP. Addressing this limitation, Serfling et al. demonstrated that ^68^Ga FAPI PET/CT yields significantly higher tumor-to-background ratios and improves the detection of occult tonsillar carcinoma compared with FDG PET/CT, thereby boosting reader confidence and facilitating primary site detection [8]. The favorable signal-to-background characteristics of FAPI are further supported by early biodistribution studies showing that normal lymphoid tissue in the palatine and lingual tonsils usually accumulates FDG, but demonstrates little, if any, FAPI activity [3,9].

These reports indicate that FAPI PET can improve the diagnostic evaluation of CUP by more accurately confirming or excluding lesions within the Waldeyer’s ring. A 2023 systematic review and meta-analysis that included 241 head and neck cancer patients reported a pooled sensitivity of 90 %, specificity of 84 %, and overall detection rate of 99 % for ^68^Ga FAPI PET, figures that compare favorably with FDG [10].

In the current case, both FDG and ^18^F-FAPI-74 showed intense uptake in the level-II neck mass, thus strengthening the suspicion of metastatic disease; however, only FDG displayed asymmetric uptake in the tonsils. The complete absence of tonsillar FAPI activity retrospectively indicates that palatine-tonsillectomy and lingual-tonsil biopsy may have been unnecessary, illustrating the potential of ^18^F-FAPI-74 PET to spare morbid diagnostic procedures.

This case further highlights a diagnostic pitfall: Branchial cleft cysts are benign congenital cystic lesions that may, at times, become inflamed and enlarge with associated pain [11]. In head and neck tumors, FNAC performs well yet is not definitive—pooled estimates are sensitivity 89.6%, specificity 96.5%, and PPV 96.2% [12], underscoring its diagnostic limitations; notably, pronounced inflammation, as seen in the present case, can further complicate the distinction between benign and malignant lesions [13]. In addition, FAP expression increases in fibrotic or chronically inflamed tissues. In the present case, pronounced fibroblastic proliferation around the branchial cleft cyst appears to explain its intense FAPI uptake, mirroring observations in pulmonary fibrosis, hepatic fibrosis, and IgG4-related disease [[5], [6], [7]]. Therefore, clinicians must cautiously interpret focal FAPI uptake within cystic or fibrotic lesions, correlating it with morphological imaging and histology as necessary.

Although ^68^Ga tracers dominate the current FAPI literature, ^18^F derivatives, such as ^18^F-FAPI-74, offer advantages in production and distribution. The 110-min half-life allows high-activity batch production at a central cyclotron and same-day shipment, while preliminary comparative studies and systematic reviews indicate comparable lesion detection between ^68^Ga- and ^18^F-labelled FAPI tracers [11,14]. These production and distribution benefits could accelerate routine clinical adoption of FAPI imaging.

This single case report cannot be used to define diagnostic accuracy; nevertheless, it underscores 2 key lessons: (i) ^18^F-FAPI-74 PET may allow improved visualization of lesions within Waldeyer’s ring compared to FDG PET, and (ii) fibrotic inflammation can yield false-positive FAPI findings. Multicenter prospective studies stratifying patients by inflammatory phenotypes are required to establish robust interpretative criteria for FAPI PET in head-and-neck oncology.

## Conclusions

In this patient, ^18^F-FAPI-74 PET/CT revealed intense uptake in a level-II cystic neck mass with no activity within Waldeyer’s ring, while FDG PET displayed asymmetric tonsillar uptake. Taken together, the FDG and FAPI findings ruled out the palatine and lingual tonsils as the primary sites. Simultaneously, strong FAPI uptake in the inflamed, fibroblast-rich branchial cleft cysts demonstrates that benign fibrotic lesions can imitate metastatic nodes. Therefore, when FAPI PET is applied in the diagnostic evaluation of suspected CUP, the absence of tracer uptake in the tonsils could be considered reassuring. Conversely, any discrete uptake in a cystic or fibrotic neck mass should prompt biopsy or other histologic confirmation rather than being accepted as a malignant disease.

## Patient consent

This case report presents findings from ongoing clinical research at Kobe City Medical Center General Hospital (UMIN000051687), which was approved by the Institutional Review Board (Approval No. 23073). The publication of this case report was conducted under the separate approval of the Institutional Review Board (Approval No. 251105). The study protocol is disclosed on our hospital’s website, providing patients the option to opt out of participation; therefore, specific written informed consent was not required for this case.
